# SOCS1: A potential diagnostic and prognostic marker for aggressive gliomas and a new target for immunotherapy

**DOI:** 10.1097/MD.0000000000040632

**Published:** 2024-12-06

**Authors:** Chuanshen Gu, Xinyi Chen, Jiayan Wu, Yiwen Zhang, Linyu Zhong, Han Luo, Wenshu Luo, Fuxia Yang

**Affiliations:** a The Fourth Clinical Medical College of Guangzhou University of Chinese Medicine, Shenzhen, Guangdong, China; b Shenzhen Traditional Chinese Medicine Hospital, Shenzhen, Guangdong, China; c The First Affiliated Hospital of Guangzhou University of Chinese Medicine, Guangzhou University of Chinese Medicine, Guangzhou, Guangdong, China; d College of Acupuncture and Tuina, Guangxi University of Chinese Medicine, Nanning, Guangxi, China.

**Keywords:** glioma, immune checkpoint, personalized prognosis model, prognostic prediction, SOCS1

## Abstract

Gliomas, the most common and deadly cancers of the central nervous system, present a unique immunological barrier that severely undermines the effectiveness of immunotherapies. Suppressor of cytokine signaling 1 (SOCS1), belonging to the SOCS protein family and playing a pivotal role in various cancer treatment strategies and is abundant in high-grade gliomas. This study conducted a comparative analysis of SOCS1 and glioma immune checkpoints. It underscores the feasibility of leveraging SOCS1 as a promising diagnostic and prognostic marker for aggressive gliomas, thus offering novel targets for glioma immunotherapy. Comprehensive gene expression analyses and clinical data validations were performed across multiple databases. The expression and biological functions of SOCS1 were examined through an array of techniques including pan-cancer analysis, functional enrichment, gene set variation analysis, and immune microenvironment examination. This was done alongside a comparison of the similarities between SOCS1 and various glioma immune checkpoints. Utilizing clinical information from patients, a bespoke predictive model was developed to further corroborate the prognostic capabilities of SOCS1. The investigation revealed considerable similarities between SOCS1 and several immune checkpoints such as CTLA4, demonstrating SOCS1’s role as an independent prognostic factor positively influencing glioma patient outcomes. The inclusion of SOCS1 in the developed predictive model significantly enhanced its precision. Our findings highlight SOCS1’s potential as an innovative target for glioma immunotherapy, providing a novel strategy to overcome the immunological barriers posed by gliomas. Furthermore, identifying SOCS1 as a viable diagnostic marker for aggressive gliomas improves the accuracy of prognostic predictions for affected patients.

## 1. Introduction

Gliomas represent the predominant form of primary malignant tumors within the adult central nervous system,^[1,2]^ notable for their aggressive growth, invasive proliferation, and propensity for recurrence. The World Health Organization (WHO) categorizes gliomas into 4 grades, among which glioblastoma (WHO IV), a variant known for its chemoresistance, comprises about 50% of all glioma incidences.^[3]^ Despite recent advances in glioma treatment modalities, the overall prognosis for the majority of patients remains grim.^[4–6]^

Immunotherapy, especially strategies involving immune checkpoint inhibitors and adoptive cell transfer, has demonstrated effectiveness against a broad range of tumors.^[7]^ Yet, in the case of high-grade gliomas, these therapies face significant challenges due to the tumors’ ability to alter the immune microenvironment. This alteration significantly suppresses the functional capacities of T cells and NK cells, thus diminishing the therapeutic impact of immunotherapeutic approaches.^[8,9]^ This has underscored the critical need to delve into the immunological landscape of gliomas, to identify and understand the dynamics of immune regulatory mechanisms, and to pinpoint immune checkpoints that are optimally suited for glioma patients, to enhance the effectiveness of immunotherapies.^[10,11]^

Research findings have suggested a positive correlation between suppressor of cytokine signaling 1 (SOCS1) expression levels and the presence of immune cells such as CD4+ T cells, neutrophils, and myeloid dendritic cells within gliomas, alongside a negative correlation with tumor purity. This positions SOCS1 as a promising candidate for novel immunotherapeutic interventions, and as a potential diagnostic and prognostic biomarker for glioma patients.^[12,13]^

No comprehensive analyses have yet been presented on SOCS1’s expression patterns and its prognostic significance within gliomas. This study seeks to bridge this gap through a pan-cancer analysis of SOCS1, investigating its expression patterns within gliomas using public single-cell sequencing data. By conducting functional enrichment analysis, both from differential expression and gene set variation analysis (GSVA) perspectives, we aim to corroborate the association between SOCS1 expression and immune functionality in glioma patients. Moreover, by examining the shared characteristics between SOCS1 and various immune checkpoints within the immune microenvironment, this research explored the feasibility of SOCS1 as a novel immunotherapeutic target. Further, it assessed the potential of SOCS1 to refine the accuracy of personalized predictive models, serving as a diagnostic and prognostic indicator for the clinical pathology and survival outcomes in glioma patients. This endeavor aims to pave new pathways for the design and enhancement of immunotherapeutic strategies against gliomas.

## 2. Materials and methods

### 2.1. Data acquisition

Transcriptomic sequencing data, alongside pertinent clinical and survival information for 693 glioma cases, were sourced from the Chinese Glioma Genome Atlas (CGGA) database (http://www.cgga.org.cn/).^[14–17]^ Additionally, the Cancer Genome Atlas (TCGA) database (https://portal.gdc.cancer.gov/) provided transcriptomic sequencing data and relevant clinical and survival details for a combined total of 702 glioma cases from the TCGA-low grade glioma (LGG) and TCGA-glioblastoma multiforme (GBM) collections. Post data cleaning, this yielded transcriptomic sequencing data and associated clinical and survival details for 650 tumor specimens and 5 samples of normal brain tissue. Single-cell sequencing data from 14 glioma patients, encompassing 6148 cells, were retrieved from the CGGA database.^[18,19]^ Single-cell sequencing data for an individual glioma case, designated as GSM5608485, was procured from the Gene Expression Omnibus (GEO) database (https://ncbi.nlm.nih.gov/geo/). Further, transcriptomic sequencing data for normal human tissue samples were obtained from both the Genotype-Tissue Expression (GTEx) project (https://www.genome.gov/Funded-Programs-Projects/Genotype-Tissue-Expression-Project) and the TCGA database.

### 2.2. Pan-cancer analysis, single-cell analysis, and differential gene expression analysis

The expression of SOCS1 in normal samples from the GTEx and TCGA databases was compared with its expression in corresponding tumor samples from the TCGA database. This comparison highlighted differential expression patterns. Additionally, public glioma single-cell sequencing data from the CGGA and GEO databases, notably GSM5608485, were used for single-cell analyses. These analyses enabled the exploration of expression discrepancies between SOCS1 and immune markers within various clusters. These clusters were derived from dimensionality reduction techniques.

Leveraging TCGA mutation data, the relationship between SOCS1 and key pan-cancer metrics, tumor mutation burden (TMB) and microsatellite instability (MSI), was assessed. Furthermore, the role of SOCS1 in pan-cancer immune infiltration was scrutinized through immune scoring.^[20]^ By grouping glioma samples from the TCGA database into categories of high and low expression based on the median expression level of SOCS1, and following stringent gene quality control and the exclusion of low-quality genes, a differential gene expression analysis was conducted using the DEG package (version R 4.3.1). Genes exhibiting a log2 fold change (log2FC) > 1 with an adjusted *P*-value < .05 were identified as upregulated, whereas those with log2FC < 1 and an adjusted *P*-value < .05 were tagged as downregulated. This process resulted in a curated list of genes differentiated by the median expression of SOCS1. Enrichment analyses for Gene Ontology (GO) and Kyoto Encyclopedia of Genes and Genomes (KEGG) pathways on this gene list were performed using Enrichplot and clusterProfiler packages (version R 4.3.1), presenting the top 5 findings for each category.

### 2.3. Functional enrichment analysis

Differential gene lists, derived from glioma samples in the CGGA and TCGA databases, were categorized based on high versus low SOCS1 expression. These lists were then analyzed to identify a cohort of SOCS1-associated genes. The respective scores of these genes were determined using the Pearson algorithm. Following selection criteria of |R|>0.5 and *P* < .05, the 203 most SOCS1-correlated genes or characteristic genes of cell clusters were submitted to the Database for Annotation, Visualization, and Integrated Discovery (DAVID, version 6.8),^[21,22]^ using official gene symbols for identification and *Homo sapiens* as the reference species. The outcomes of GO and KEGG pathway enrichments were obtained, and gene set enrichment analysis (GSEA) was carried out on the differential gene lists with the GSEA package (version R 4.3.1), showcasing the leading 5 entries organized by ascending *P*-value (*P* < .05).

### 2.4. Gene set variation analysis

We acquired lists of genes involved in human immune processes and inflammatory responses from the AmiGO 2 portal (http://amigo.geneontology.org/amigo). Employing the GSVA package (version R 4.3.1) with default settings, we systematically computed functional enrichment scores for glioma samples within the CGGA and TCGA databases. Visualization of the enrichment outcomes was achieved through heatmaps, crafted using the pheatmap package (version R 4.3.1). Through Pearson correlation analysis, we explored the association between SOCS1 expression and the activities of the immune system and inflammation. We determined the correlation coefficients (Coef) between SOCS1 and specific genes, as well as gene sets linked to immune and inflammatory functions. The findings were then presented.

### 2.5. Immune infiltration and TMB analysis

By differentiating glioma samples into high and low expression groups based on the median SOCS1 expression value, immune scores and tumor purity were determined for samples from the CGGA and TCGA databases, respectively. Utilizing the GSVA framework, the degree of tumor immune cell infiltration was assessed through the single-sample gene set enrichment analysis (ssGSEA) algorithm, complemented by cell type enrichment analysis via the xCell algorithm. Furthermore, CIBERSORT was employed to estimate the abundance of immune cells, investigating the correlations between SOCS1, certain immune checkpoints, immune scores, cell types, and immune cells. TMB analyses were performed leveraging mutation data from glioma samples in the TCGA database.

### 2.6. Immune checkpoint and drug sensitivity analysis

Glioma samples from the CGGA and TCGA databases were stratified into high and low SOCS1 expression groups based on the median expression level of SOCS1. The expression levels of 3 immune checkpoints: PDCD1, CD274, and PDCD1LG2, were subsequently analyzed within these groups. Furthermore, the differential sensitivity to 2 prominent antitumor drugs across the groups with varying SOCS1 expression levels was investigated using data from the genomics of drug sensitivity in cancer ^[23]^ and the Cancer Therapeutics Response Portal^[24–26]^ databases.

### 2.7. Association of SOCS1 expression with clinical characteristics in glioma

Patients with incomplete survival data were excluded from the analysis. Subsequently, the R programming language facilitated the visualization of differential expression of SOCS1 across clinical features in glioma patients within the CGGA and TCGA databases. An evaluation was conducted to ascertain the association between SOCS1 expression and a spectrum of clinical characteristics. The clinical characteristics assessed included WHO tumor grades, chromosomal 1p19q co-deletion status, mutations in the gene encoding isocitrate dehydrogenase (IDH), promoter methylation status (MGMT), and patient age.

### 2.8. Statistical analysis

Statistical analysis and visualization of glioma patient data from the CGGA and TCGA databases were performed using R (https://www.r-project.org/, version 4.3.1), IBM SPSS Statistics (version 26.0), and GraphPad Prism (version 9.5). The significance of between-group variation was determined using unpaired *t* tests, whereas one-way Analysis of Variance was applied for comparisons among multiple groups. The significance of correlation Coef between the 2 groups were verified using Pearson correlation analysis. Continuous variables were analyzed with the Wilcoxon rank-sum test, while categorical variables were evaluated using the chi-square test. To assess SOCS1’s diagnostic performance for patient pathological features and its prognostic potential, we plotted Receiver Operating Characteristic (ROC) curves and time-dependent ROC curves. Kaplan–Meier survival curves illustrated the survival distributions for groups with high versus low SOCS1 expression, and both univariate and multivariate Cox proportional hazards models were used to evaluate SOCS1’s prognostic significance. Baseline characteristic tables were employed to present the distribution and proportion of patient clinical feature groups. Patients lacking clinical or survival data were excluded from relevant analyses. A *P*-value of <.05 was considered statistically significant, and all tests were two-tailed.

### 2.9. LASSO-COX analysis

We utilized the glmnet and survival packages (version R 4.3.1) for a LASSO-COX dimensionality reduction analysis on TCGA glioma samples, categorizing them by the median expression of SOCS1 to isolate differential genes. Based on the candidate genes and their associated regression Coef, we calculated a risk score for each patient. Patients were subsequently divided into high and low-risk groups according to the median risk score, to compare risk scores across varying survival states. ROC curves were drawn to test the fidelity of the risk score in forecasting patient survival outcomes, with survival time and risk score depicted as dependent variables in a risk distribution plot. The robustness of this LASSO-COX model was further tested using glioma samples from the CGGA database.

### 2.10. Nomogram model development

For glioma samples in the TCGA database, a multifactorial Cox regression analysis was carried out on clinical characteristics. This analysis led to the creation of a Nomogram model that includes MGMT, age, grade, IDH, 1p19q, and risk factors. The model’s differentiation capability and the significance of incorporated factors were quantified using the C-index, calculated via the rms package (version R 4.3.1). The model’s precision in forecasting survival was evaluated with calibration curves and the C-index, with validation conducted on glioma samples from the CGGA database.

## 3. Results

### 3.1. High expression of SOCS1 in gliomas

**Figure 1.**
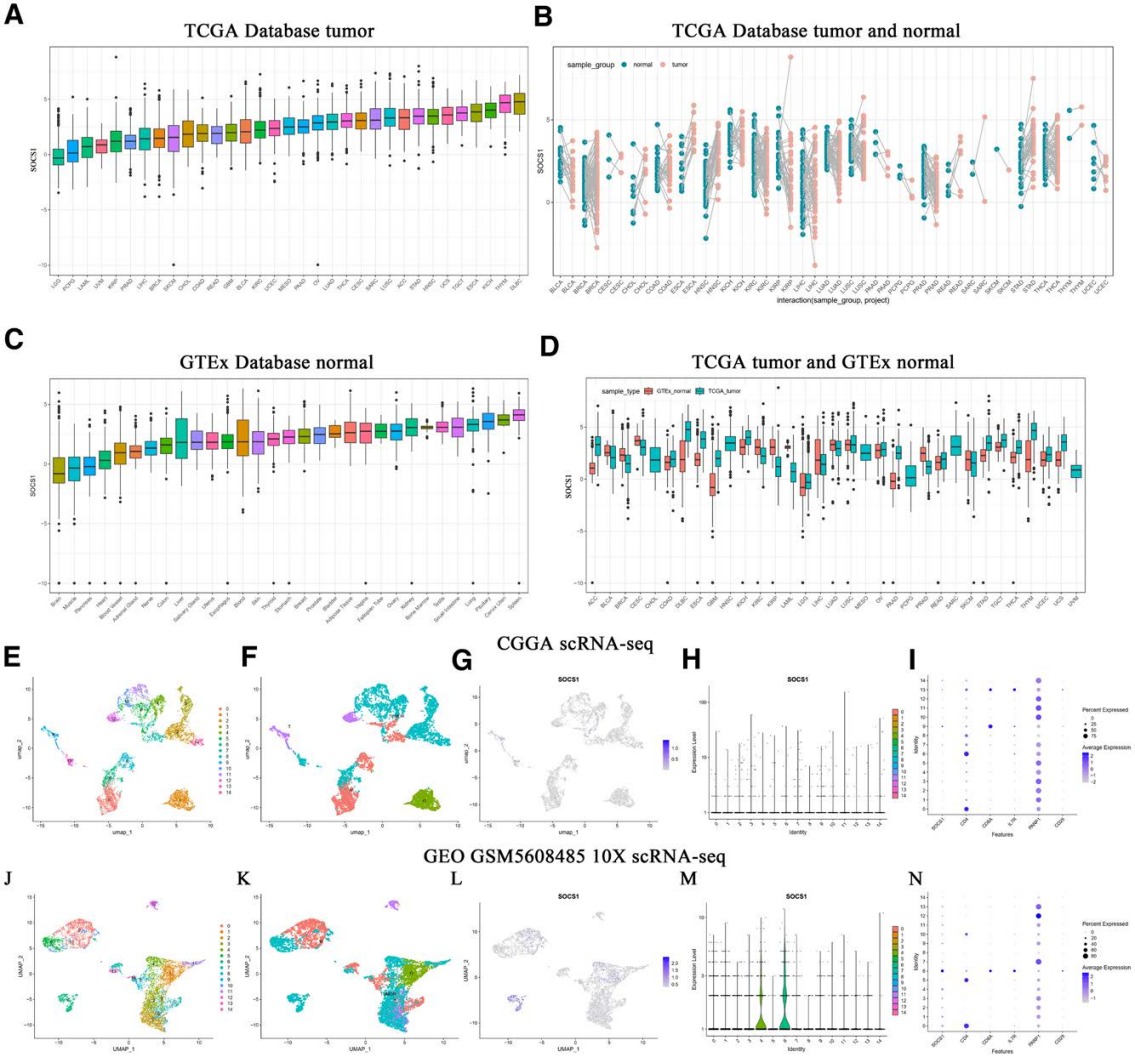
Disparities in SOCS1 expression across pan-cancer and analysis of public glioma single-cell sequencing data: (A) SOCS1 expression across pan-cancer in the TCGA database. (B) The expression of SOCS1 in pan-cancer and normal paired samples in the TCGA database. (C) SOCS1 expression in normal samples within the GTEx database. (D) Expression of SOCS1 in pan-cancer and normal paired samples from the TCGA database and the GTEx database. (E and J) UMAP clustering of glioma single-cell sequencing data from CGGA and GEO (GSM5608485). (F and K) Annotations based on clustering genes: TUMOR for tumor cells, T for T cells, O for oligodendrocytes, M for macrophages. (G and L) Expression of SOCS1 in each cluster. (H and M) Group-specific SOCS1 expression. (I and N) Expression of SOCS1 and selected immune markers in each group. CGGA = Chinese Glioma Genome Atlas, GEO = Gene Expression Omnibus, GTEx = genotype-tissue expression, SOCS1 = suppressor of cytokine signaling 1, TCGA = the Cancer Genome Atlas, UMAP = Uniform Manifold Approximation and Projection.

Pair-wise comparisons between normal and tumor samples within the TCGA database revealed SOCS1 to be highly expressed in BRCA, ESCA, HNSC, LIHC, LUAD, LUSC, READ, STAD, and THCA, and it exhibits low expression in BLCA, CESC, COAD, KIRC, PRAD, and UCEC (Fig. 1A and B). When comparing normal samples from the GTEx database against tumor samples from the TCGA database, SOCS1 was found to be overexpressed in ACC, COAD, DLBC, ESCA, GBM, KICH, LGG, PAAD, READ, STAD, TGCT, THCA, THYM, UCEC, and UCS, but underexpressed in BLCA, BRCA, CESC, KIRC, KIRP, LIHC, LUAD, PRAD, and SKCM (Fig. 1C and D).

### 3.2. SOCS1 expression distribution in gliomas resembles that of certain immune markers

To delve deeper into SOCS1’s expression within gliomas, we analyzed single-cell sequencing data from the CGGA and GEO databases (GSM5608485) using the Seurat package (version R 4.3.1). This analysis identified that SOCS1 expression is predominantly found in clusters 4, 6, 9, and 14, showing high levels in tumor cells and T cells, with moderate a expression in oligodendrocytes and macrophages (Fig. 1E–H, J–M). A comparative analysis of SOCS1’s expression profiles with those of 5 immune markers revealed similarities between SOCS1 and CD8A, IL7R (Fig. 1I and N).

### 3.3. Correlation between SOCS1 expression and TMB, MSI, and immune scores in certain cancers

**Figure 2.**
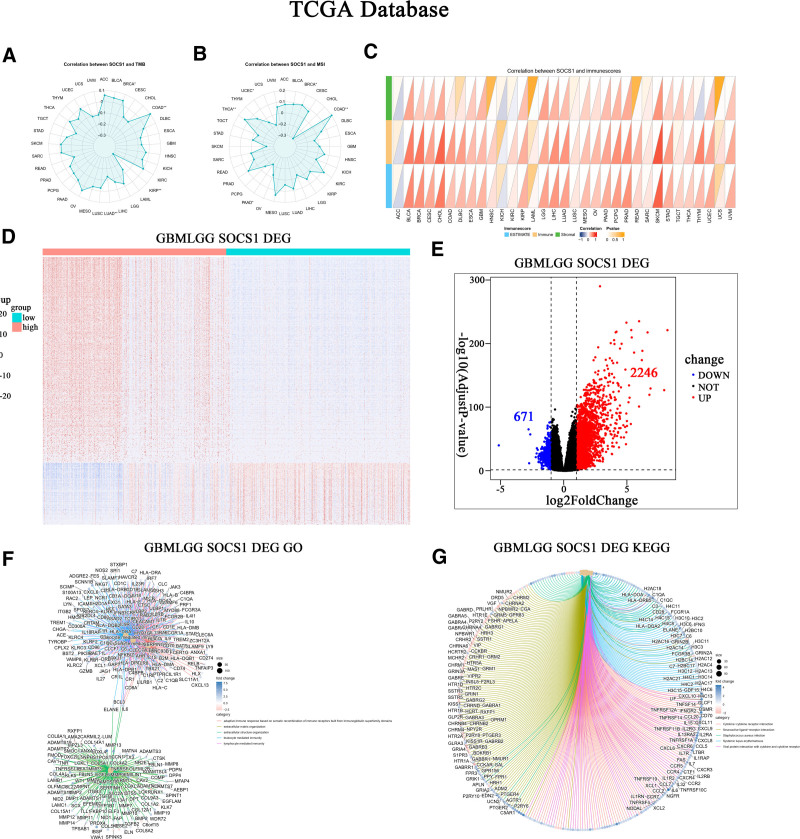
Correlation of SOCS1 with pan-cancer TMB, MSI, and immune scores and differential gene expression analysis in TCGA glioma samples. (A) Radar chart illustrating the correlation between SOCS1 expression and pan-cancer TMB in the TCGA database, with most results showing no significant differences, *P < .05, **P < .01. (B) Radar chart illustrating the correlation between SOCS1 expression and pan-cancer MSI in the TCGA database, with most results showing no significant differences, *P < .05, **P < .01. (C) The correlation between SOCS1 and pan-cancer immune scores in the TCGA database depicted using a triangular representation. (D) Heatmap of the differential gene expression analysis between high and low SOCS1 expression groups in TCGA glioma samples. (E) Identification of 671 downregulated genes (log2FC < 1, adjusted *P* < .05) and 2246 upregulated genes (log2FC > 1, adjusted *P* < .05), displayed in a volcano plot. (F) Dandelion plot showcasing GO enrichment analysis for genes differentially expressed between high and low SOCS1 expression. (G) Network diagram for KEGG pathway enrichment analysis of genes differentially expressed between high and low SOCS1 expression. KEGG = Kyoto Encyclopedia of Genes and Genomes, SOCS1 = suppressor of cytokine signaling 1, TCGA = the Cancer Genome Atlas, TMB = tumor mutation burden.

The correlation between SOCS1 expression with pan-cancer TMB and MSI was assessed using data from the TCGA database. Results indicate a positive correlation between SOCS1 expression and TMB in cancers like COAD and BRCA, while a negative correlation was observed in LUAD and KIRP (Fig. 2A). Similarly, SOCS1 expression showed a positive correlation with MSI in COAD and THCA, but a negative correlation in PAAD and UCEC (Fig. 2B). An initial exploration into the relationship between SOCS1 expression and pan-cancer immune scores revealed a positive correlation with the immune scores of a majority of cancers, including CHOL, LGG, and GBM. These findings were statistically significant (Fig. 2C).

### 3.4. SOCS1 modulates immune response via an immune receptor cell-dependent mechanism

The TCGA glioma samples to analyze differences in gene expression in the database, the sample is divided into SOCS1 high expression and low expression group. This analysis identified 671 downregulated genes and 2246 upregulated genes (Fig. 2D and E). Enrichment analyses of these differential genes in GO and KEGG pathways revealed a significant enrichment in functions related to adaptive immune responses facilitated by immune receptor cell recombination and immune responses mediated by leukocytes and lymphocytes. Enriched pathways predominantly involved cytokine-receptor interactions, neuroactive ligand–receptor interactions, viral protein–cytokine interactions, and cytokine–cytokine interactions (Fig. 2F and G).

### 3.5. SOCS1’s links to tumor immunity and inflammatory responses

**Figure 3.**
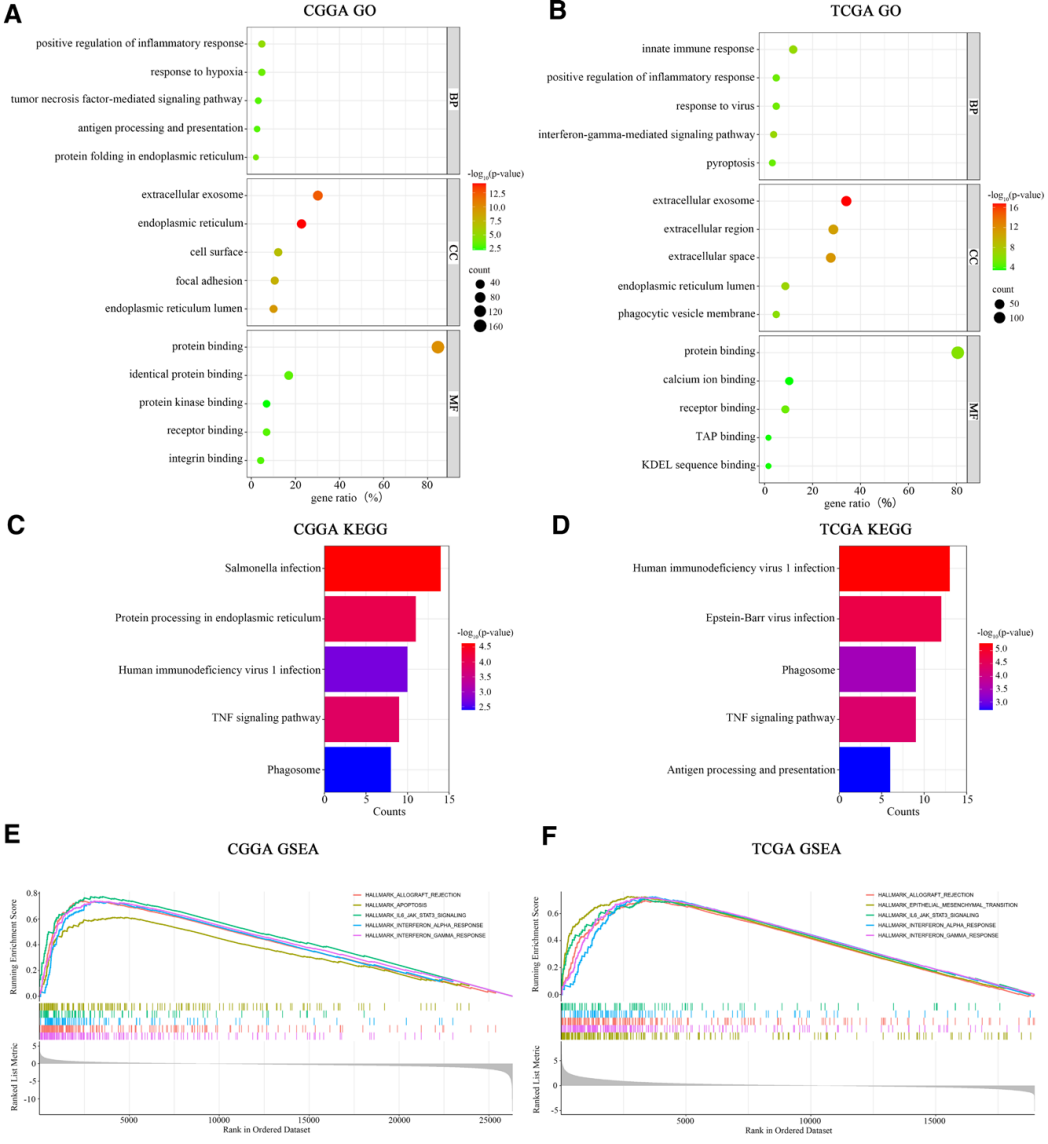
Functional enrichment analysis of SOCS1-associated genes in glioma samples from CGGA and TCGA databases. Differential gene sequences obtained from analyses of high and low SOCS1 expression groups in glioma samples underwent Pearson correlation analysis to extract SOCS1-associated gene sets and scores (|*R*| >0.5, *P* < .05). The 203 genes or cell cluster characteristic genes most strongly associated with SOCS1 were chosen for GO enrichment analysis (A and B). KEGG pathway enrichment analysis (C and D); GSEA enrichment analysis (adjusted *P* < .05, FDR < 0.25) (E and F). CGGA = Chinese Glioma Genome Atlas, GSEA = gene set enrichment analysis, SOCS1 = suppressor of cytokine signaling 1, TCGA = the Cancer Genome Atlas.

To further explore the biological functions associated with SOCS1 in glioma patients, GO and KEGG enrichment analyses were conducted on gene sets correlated with SOCS1. These analyses revealed several key findings. In glioma samples from the CGGA database, the biological processes most closely associated with SOCS1 include 3 main areas. These areas are the positive regulation of inflammatory responses, tumor necrosis factor-mediated signal transduction, and antigen processing and presentation. The cellular components mainly involved are found in exosomes, the endoplasmic reticulum, and on the cell surface, with the principal molecular functions being protein binding and protein kinase binding. The principal pathways identified involve protein processing within the endoplasmic reticulum, TNF signaling, and reactions to infections by Salmonella and HIV (as shown in Fig. 3A and C). In the case of glioma samples obtained from the TCGA database, the biological processes most strongly linked to SOCS1 encompass innate immune responses, the positive regulation of inflammatory responses, and viral response mechanisms, with cellular components predominantly located in exosomes and intercellular spaces, and the signaling pathways mirroring those identified in the CGGA database (Fig. 3B and D). GSEA enrichment analysis pinpointed the signaling pathways implicating SOCS1 in glioma patients, indicating that high SOCS1 expression in the CGGA glioma samples correlates with allograft rejection, programmed cell death, IL-6 and JAK-STAT3 signaling pathways, and interferon-gamma responses (Fig. 3E). The signaling pathways associated with high SOCS1 expression in the TCGA glioma samples showed similarity to those in the CGGA database (Fig. 3F).

### 3.6. Activation of established cancer immune checkpoint inhibitors and inflammation dysregulation positively correlate with high SOCS1 expression

**Figure 4.**
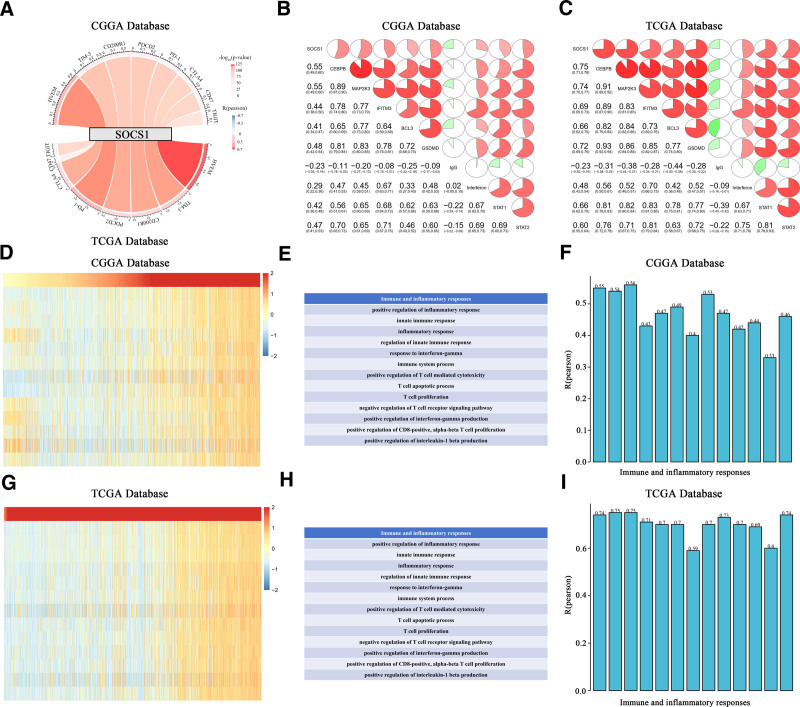
Correlation study of SOCS1 expression with immune pathways and inflammatory activity in glioma samples from the CGGA and TCGA databases. (A) The Pearson correlation between SOCS1 and various inhibitory immune checkpoints, where band width represents the *R*-value and color indicates the *P*-value. (B and C) Correlation matrices between SOCS1 and meta-gene clusters related to immunity or inflammation, with proportions in the pie charts indicating positive (red) and negative (green) correlations, analyzed using Pearson correlation. (D and G) Analysis of the correlation between SOCS1 expression and immune function enrichment scores, represented in heatmaps that align SOCS1 expression with the enrichment scores of immune functions for each patient in the glioma samples, sorted by increasing SOCS1 expression. The table to the right lists the names of functions tested, and the bar charts show average *R*-values from the correlation analysis (*P* < .05). CGGA = Chinese Glioma Genome Atlas, SOCS1 = suppressor of cytokine signaling 1, TCGA = the Cancer Genome Atlas.

By conducting Pearson correlation analysis on glioma samples from the CGGA and TCGA databases, it was discovered that the expression of SOCS1 is significantly positively correlated with several known inhibitory immune checkpoints, including HVEM, TIM-3, PD-1, PDCD2, TIGIT, CD200R1, CTLA4, and CD47. This correlation suggests that high SOCS1 expression contributes to the suppression of immune responses against gliomas (Fig. 4A). Moreover, we investigated the relationship between SOCS1 and 9 meta-gene clusters related to immune systems or inflammatory responses, such as CEBPB, MAP2K3, IFITM3, BCL3, GSDMD, IgG, interferons, and STAT1/2. The examination revealed a notably positive correlation between the expression of SOCS1 and the majority of these markers related to immunity and inflammation (Fig. 4B and C).

Within the framework of immunogenic cancer cell death, the stimulation of lymphocytes (such as NK, T, and B cells) along with the secretion of chemokines and cytokines, can amplify the apoptosis or necrosis of tumor cells. Therefore, to assess the impact of SOCS1 activation on immune pathways and cytokine profiles in glioma patients, GSVA analysis was employed to calculate the enrichment scores for immune-related processes in glioma samples from the CGGA and TCGA databases. The association between these scores and SOCS1 expression reveals a positive link between SOCS1 expression and the majority of immune activities and inflammatory reactions. This association contributes to the growth and programmed cell death of T cells and CD8 cells in gliomas (Fig. 4D–I).

### 3.7. High expression of SOCS1 in gliomas positively correlates with immune scores

**Figure 5.**
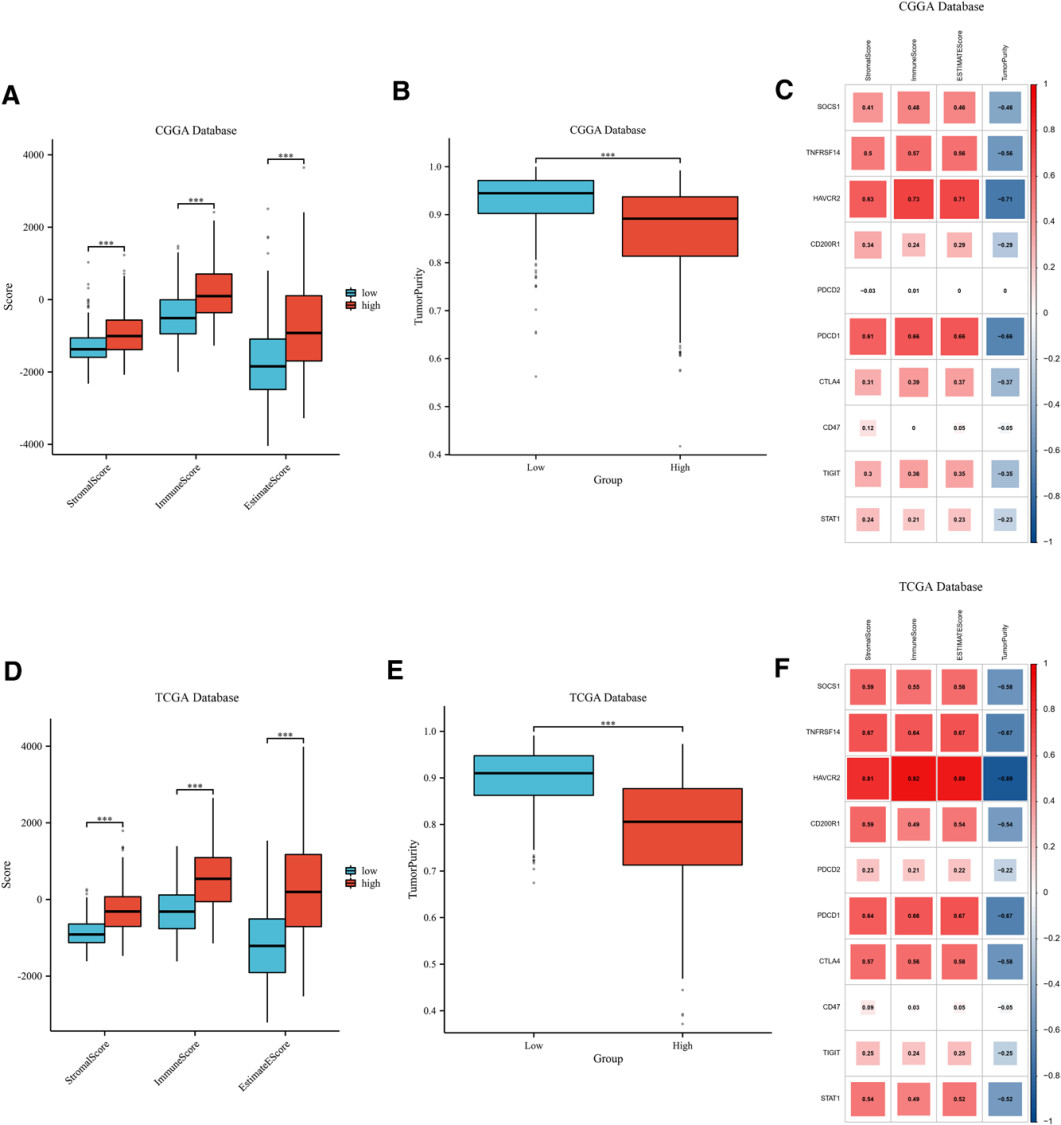
Correlation analysis of SOCS1 expression with immune scores in glioma samples from the CGGA and TCGA databases. (A and D) Box plots illustrate the correlation between high and low SOCS1 expression groups in glioma samples with their stromal, immune, and ESTIMATE scores, ***P < .001. (B and E) Box plots reveal the correlation between high and low SOCS1 expression groups in glioma samples and tumor purity, ***P < .001. (C and F) Correlation matrices show the associations between SOCS1 and 9 immune checkpoint expressions with stromal, immune, ESTIMATE scores, and tumor purity in glioma patients, where red signifies positive correlations and blue indicates negative correlations. CGGA = Chinese Glioma Genome Atlas, SOCS1 = suppressor of cytokine signaling 1, TCGA = the Cancer Genome Atlas.

Stromal, immune, and ESTIMATE scores, and tumor purity, were assessed for glioma samples classified into high and low SOCS1 expression groups within the CGGA and TCGA databases. The results show that the group with lower SOCS1 expression had reduced stromal, immune, and ESTIMATE scores compared to the group with higher expression, implying a decreased tumor purity in those with elevated SOCS1 expression (Fig. 5A, B and D, E). Additionally, the relationship between SOCS1 and 9 immune checkpoint expressions and stromal, immune, ESTIMATE scores, and tumor purity in glioma patients was analyzed. The analysis revealed that the expression of SOCS1 and most immune checkpoints positively correlates with stromal, immune, and ESTIMATE scores, while inversely correlating with tumor purity (Fig. 5C and F).

**Figure 6.**
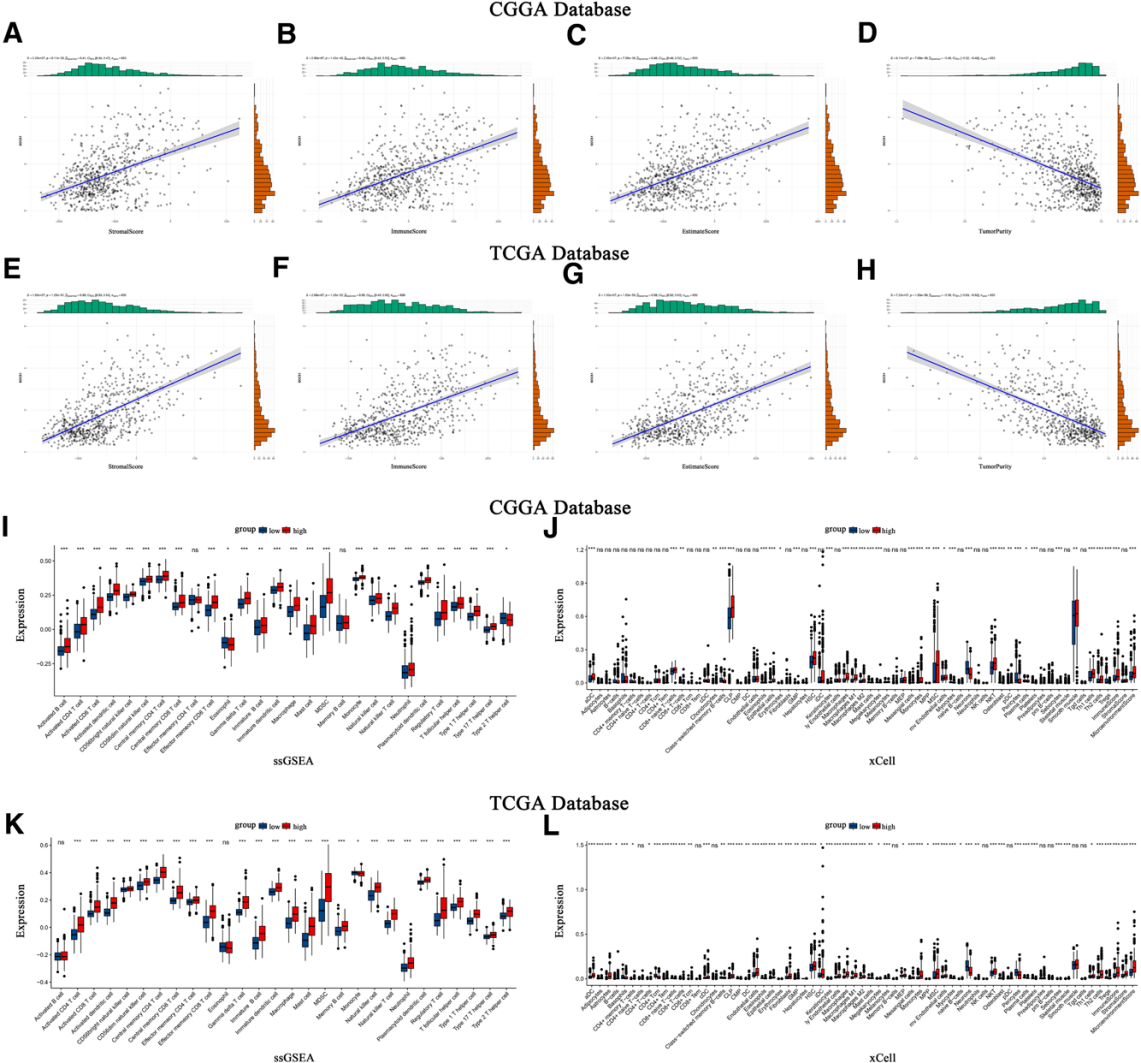
CGGA and TCGA database glioma samples analysis of SOCS1 Expression correlation with immune scores, and ssGSEA and Xcell enrichment analyses. (A–H) Scatter plots correlating SOCS1 expression with stromal scores, immune scores, ESTIMATE scores, and tumor purity. (I and K) Immune cell infiltration levels in high and low SOCS1 expression groups assessed by ssGSEA, ns *P* > .05, *P < .05, **P < .01, ***P < .001. (J and L) Infiltration scores for 64 types of immune and stromal cells in high and low SOCS1 expression groups calculated using Xcell, ns *P* > .05, *P < .05, **P < .01, ***P < .001. CGGA = Chinese Glioma Genome Atlas, SOCS1 = suppressor of cytokine signaling 1, TCGA = the Cancer Genome Atlas.

Scatter plots illustrate the correlation between SOCS1 expression and stromal scores, immune scores, ESTIMATE scores, and tumor purity in glioma patients (Fig. 6A–H). The ssGSEA method assessed immune cell infiltration in glioma samples from the CGGA and TCGA databases, categorized by SOCS1 expression levels. It founds that the high SOCS1 expression group had greater immune cell infiltration compared to the low expression group. Cell type enrichment analysis, conducted with the Xcell algorithm, indicated that the same cell types had higher scores in the high SOCS1 expression group than in the low expression group (Fig. 6I–L).

### 3.8. SOCS1 shares similarities with most immune checkpoints

**Figure 7.**
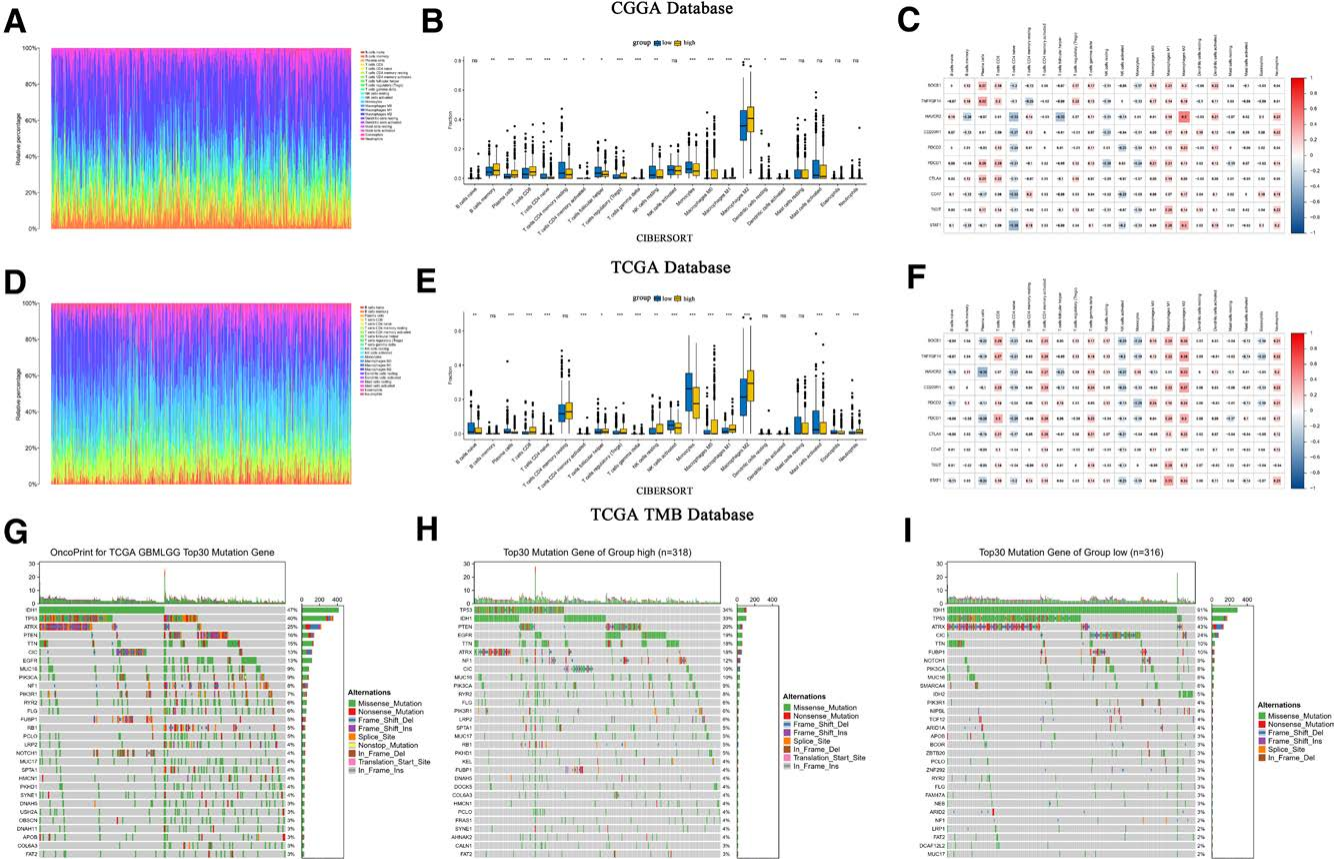
Analysis of immune cell abundance in high and low SOCS1 expression groups of glioma samples from the CGGA and TCGA databases, and TMB analysis using glioma mutation data from the TCGA database. (A and D) Rainbow charts showcase the immune cell abundance estimated by CIBERSORT in glioma samples with high and low SOCS1 expression. (B and E) Box plots depict the proportions of 22 types of immune cells in high and low SOCS1 expression groups, calculated by CIBERSORT, with ns indicating *P* > .05, * *P* < .05, ** *P* < .01, and *** *P* < .001. (C and F) A correlation matrix displays the associations between SOCS1, 9 immune checkpoints, and 22 immune cell types, where red symbolizes positive correlations and blue denotes negative ones. (G) A waterfall chart for TMB analysis using glioma mutation data from the TCGA database highlights the top 30 most mutated genes. (H and I) Mutation data divided by the median SOCS1 expression into high and low groups, with waterfall charts presenting the top 30 mutations for each group. CGGA = Chinese Glioma Genome Atlas, SOCS1 = suppressor of cytokine signaling 1, TCGA = the Cancer Genome Atlas, TMB = tumor mutation burden.

CIBERSORT estimated the abundance and proportion of 22 types of immune cells in glioma samples from the CGGA and TCGA databases, comparing high and low SOCS1 expression groups. The analysis explored the correlation of SOCS1 with 9 immune checkpoints and 22 types of immune cells. In the CGGA database, plasma cells, T cells CD8, monocytes, and macrophages M2 exhibited high and significantly different proportion scores. Among these, M2 macrophages scored the highest. Specifically, the proportion scores of T cells CD8 and M2 macrophages were higher in the high SOCS1 expression group (Fig. 7A and B). Correlation analysis revealed SOCS1 positively correlated with B cells memory, plasma cells, T cells CD8, T cells regulatory, T cells gamma delta, macrophages M0, macrophages M1, macrophages M2, and dendritic cells activated, aligning closely with the correlation analysis of CTLA4 with 22 types of immune cells (Fig. 7C). Validation was performed in the TCGA database using the same methodology, yielding similar results (Fig. 7D and E). However, SOCS1 was only positively correlated with CD8 T cells, T cells CD4 memory resting, T cells CD4 memory activated, T cells follicular helper, T cells regulatory, T cells gamma delta, NK cells resting, macrophages M0, macrophages M2, macrophages M3 macrophages, dendritic cells activated, and neutrophils in the correlation analysis, showing decreased similarity with CTLA4 correlation but maintaining similarity with more immune checkpoint analyses (Fig. 7F).

TMB analysis, leveraging glioma mutation data from the TCGA database, illustrating the 30 genes most frequently mutated (Fig. 7G). Additionally, mutation data were categorized into groups of high and low SOCS1 expression, determined by the median expression of SOCS1, revealing the top 30 mutated genes within each category (Fig. 7H and I). The findings showed that IDH1 and TP53 were the most frequently mutated genes, and the tumor mutation burden was slightly greater in the group with higher SOCS1 expression compared to the group with lower expression.

### 3.9. Immune checkpoint blockade may offer enhanced efficacy in the high SOCS1 expression group

**Figure 8.**
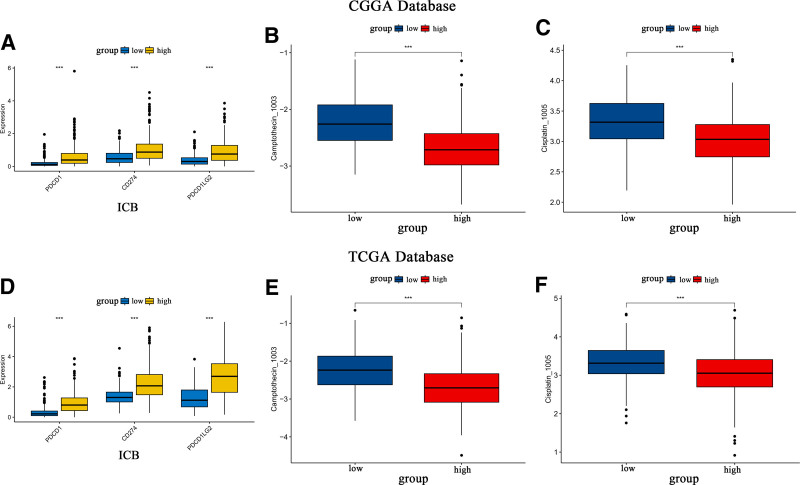
Immune checkpoint blockade testing and drug sensitivity analysis in high and low SOCS1 Expression groups of glioma samples from the CGGA and TCGA databases. (A and D) Box plots for the disparity in responses to immune checkpoint blockade between high and low SOCS1 expression groups for PDCD1, CD274, and PDCD1LG2 in glioma samples, ***P < .001 indicating significant differences. (B and E) Box plots for drug sensitivity for the antitumor drug Camptothecin_1003 across high and low SOCS1 expression groups, ***P < .001 denoting significant sensitivity. (C and F) Box plots reveal drug sensitivity for the antitumor drug Cisplatin_1005 in high and low SOCS1 expression groups, ***P < .001. CGGA = Chinese Glioma Genome Atlas, SOCS1 = suppressor of cytokine signaling 1.

Assessments of the immune blockade response to 3 checkpoints (PDCD1, CD274, and PDCD1LG2) and the IC50 for 2 classical antitumor drugs were conducted for glioma samples with varying SOCS1 expression levels in the CGGA and TCGA databases. The outcomes revealed that the high SOCS1 expression group exhibited significantly stronger immune checkpoint blockade responses and heightened sensitivity to classic antitumor medications when compared to the low expression group (Fig. 8A–F). These findings imply that the high SOCS1 expression group may benefit more from immune therapy and require lower concentrations of therapeutic drugs.

### 3.10. SOCS1 is enriched in gliomas with a higher malignancy grade

**Figure 9.**
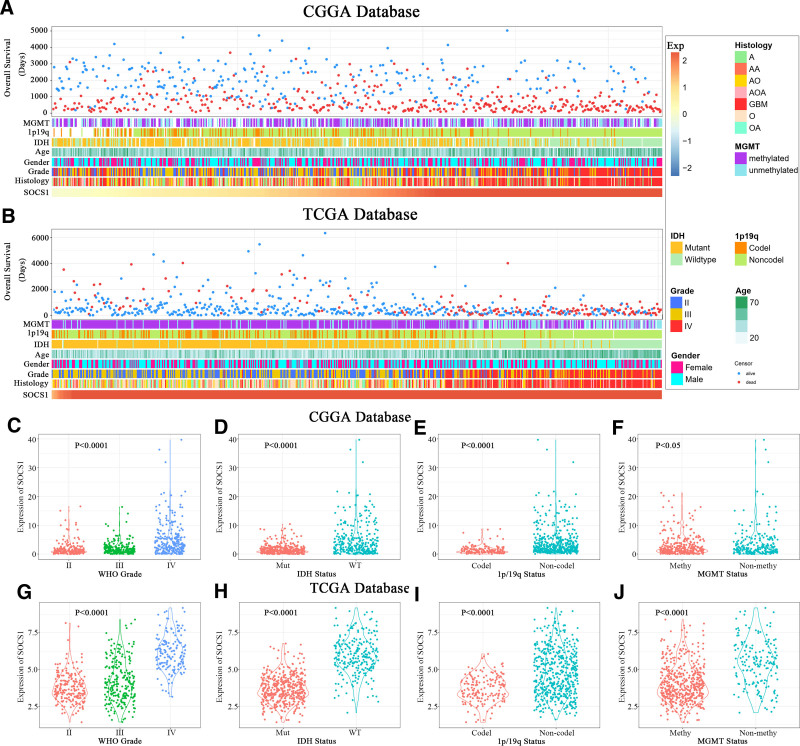
Relationship between SOCS1 and clinical pathological features in gliomas. (A) Overview of SOCS1-related clinical pathological features in gliomas from the CGGA database. (B) Overview of SOCS1-related clinical pathological features in gliomas from the TCGA database. (C and G) SOCS1 significantly increases in high-grade gliomas in both the CGGA and TCGA databases, with differences assessed using one-way ANOVA. (D and H) A significant increase in SOCS1 in gliomas without IDH mutation in both the CGGA and TCGA databases, with differences assessed using unpaired *t*-tests. (E and I) A significant increase in SOCS1 in gliomas without 1p/19q co-deletion in both the CGGA and TCGA databases, assessed using unpaired *t* tests. (F and J) An increase in SOCS1 expression in gliomas lacking MGMT promoter methylation, with differences evaluated using unpaired *t* tests. ANOVA = Analysis of Variance, CGGA = Chinese Glioma Genome Atlas, IDH = isocitrate dehydrogenase, SOCS1 = suppressor of cytokine signaling 1, TCGA = the Cancer Genome Atlas.

Patients with varying levels of SOCS1 expression display distinct clinical and pathological features. The distribution of increased SOCS1 expression, alongside MGMT promoter methylation status, 1p/19q co-deletion status, IDH mutation status, and WHO grading, was observed asymmetrically in both CGGA and TCGA datasets (Fig. 9A and B). Comparative analyses within these groups demonstrated that SOCS1 expression was significantly enriched in high-grade gliomas (Fig. 9C and G) and gliomas with wild-type IDH (Fig. 9D and H) within the CGGA database. Moreover, samples lacking 1p/19q co-deletion (Fig. 9E and I) and those without MGMT promoter methylation (Fig. 9F and J) exhibited higher SOCS1 expression levels. These observations were corroborated in the TCGA dataset, indicating a prevalence of SOCS1 in more malignant forms of gliomas.

### 3.11. SOCS1 exhibits significant prognostic value for glioma patients

**Figure 10.**
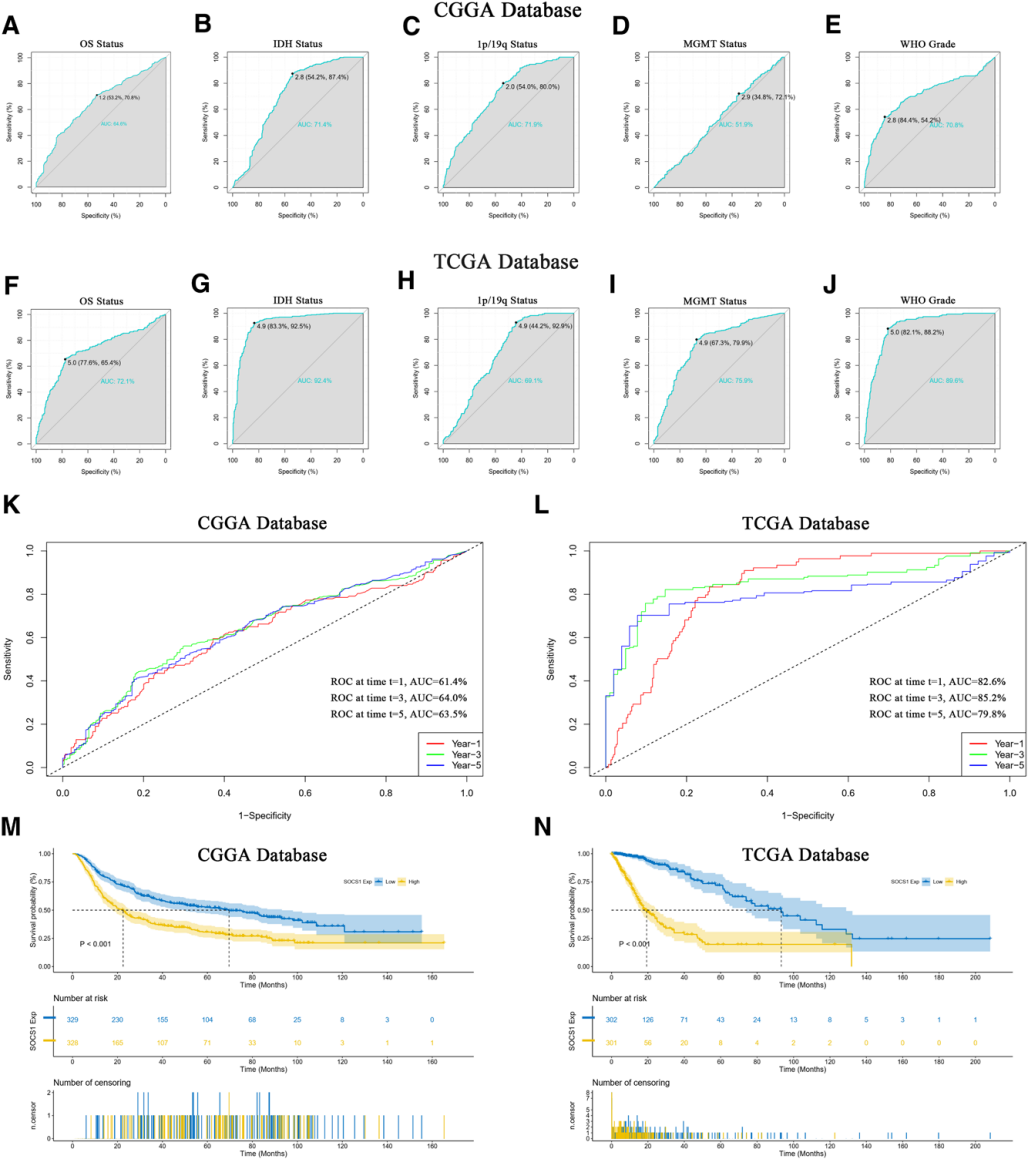
Survival status and clinical feature prediction analysis using SOCS1 in glioma patients. (A–J) ROC curve evaluations of SOCS1’s predictive performance for glioma patient survival and clinical characteristics, with the AUC indicating prediction accuracy. For ROC analysis of SOCS1 expression across glioma grades, grade II and III were classified as low-grade, and grade IV as high-grade, according to WHO standards. (K and L) Time ROC curve assessments of SOCS1’s accuracy in predicting glioma patient survival outcomes, highlighting the AUC at 1, 3, and 5 years in both databases. (M and N) Kaplan–Meier curve analysis of SOCS1 expression in the CGGA and TCGA databases, using the median expression of SOCS1 as the division threshold, with prognostic significance tested through log-rank tests. AUC = area under the curve, CGGA = Chinese Glioma Genome Atlas, ROC = receiver operating characteristic, SOCS1 = suppressor of cytokine signaling 1, TCGA = the Cancer Genome Atlas.

The prognostic value of SOCS1 in predicting glioma patient survival status and clinical features was evaluated using ROC curves (Fig. 10A–J). Specifically, the AUC for IDH mutation status in the CGGA database was 71.4% (Fig. 10B), while in the TCGA database, it reached an impressive 92.4% (Fig. 10G). The AUC for WHO grading was 70.8% in the CGGA database (Fig. 10E) and soared to 89.6% in the TCGA database (Fig. 10J). These results indicate SOCS1 could act as a predictive biomarker for IDH mutation status and high-grade gliomas. Time ROC curves further validated the efficacy of SOCS1 in forecasting glioma patient outcomes, showing the highest accuracy at the third year with an AUC of 64.0% in the CGGA database (Fig. 10K) and 85.2% in the TCGA database (Fig. 10L), indicating peak predictive precision at this timeline. To delve into the prognostic potential of SOCS1 in glioma patients, Kaplan–Meier curve analyses were conducted utilizing samples from the CGGA and TCGA databases. It was observed that patients in the CGGA database with high SOCS1 expression had significantly shorter overall survival compared to those with low expression, indicating a higher survival probability for patients with lower SOCS1 levels (Fig. 10M). This observation was corroborated in the TCGA database (Fig. 10N).

### 3.12. SOCS1 as a positive independent prognostic indicator for glioma patients

**Figure 11.**
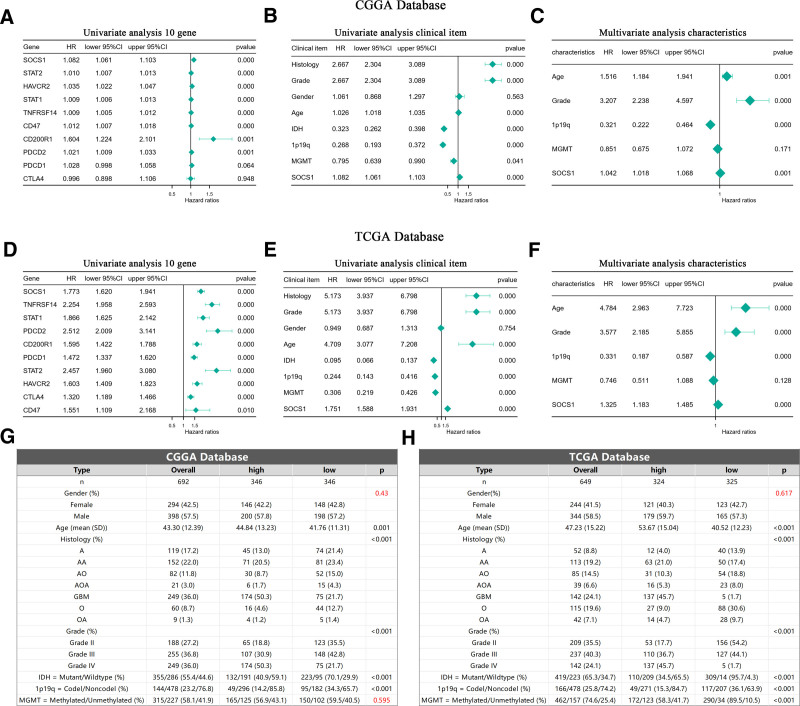
Univariate and multivariate regression analyses of SOCS1 with clinical pathological characteristics of glioma patients and corresponding baseline tables. (A and D) Univariate analysis of SOCS1 and 9 immune checkpoints with overall survival (OS) in the CGGA and TCGA databases. (B and E) Univariate analysis of prognostic factors with OS in the CGGA and TCGA databases. (C and F) Multivariate analysis of prognostic factors with OS in the CGGA and TCGA databases. (G and H) Baseline tables of glioma patient data in the CGGA and TCGA databases. Death was defined as a positive outcome, survival as a negative outcome, HR > 1 indicates a positive prognostic factor, HR < 1 indicates a negative prognostic factor. CGGA = Chinese Glioma Genome Atlas, CI = confidence interval, HR = hazard ratio, IDH = isocitrate dehydrogenase, SOCS1 = suppressor of cytokine signaling 1, TCGA = the Cancer Genome Atlas, WHO = World Health Organization.

Analysis using Cox proportional hazards models on glioma samples from the CGGA and TCGA databases initially established a univariate model. This model included SOCS1 and 9 immune checkpoints to evaluate their potential as independent prognostic factors for glioma patients. The analysis revealed that SOCS1, along with 7 of the immune checkpoints, qualifies as independent prognostic factors. Notably, SOCS1 exhibited a higher hazard ratio among the genes examined (Fig. 11A and D). Subsequent incorporation of SOCS1 into both univariate and multivariate Cox proportional hazards models, alongside clinical characteristics of glioma patients, demonstrated that SOCS1 acts as a positive prognostic factor in the univariate analysis (Fig. 11B and E). Moreover, multivariate analysis confirmed SOCS1 as a positive prognostic factor independent of established prognostic indicators such as WHO grading, age at diagnosis, 1p/19q co-deletion status, and MGMT promoter methylation status (Fig. 11C and F). Baseline tables derived from patient clinical data lend further support to these findings (Fig. 11G and H).

### 3.13. Risk scoring based on differential expression SOCS1 showcases high credibility

**Figure 12.**
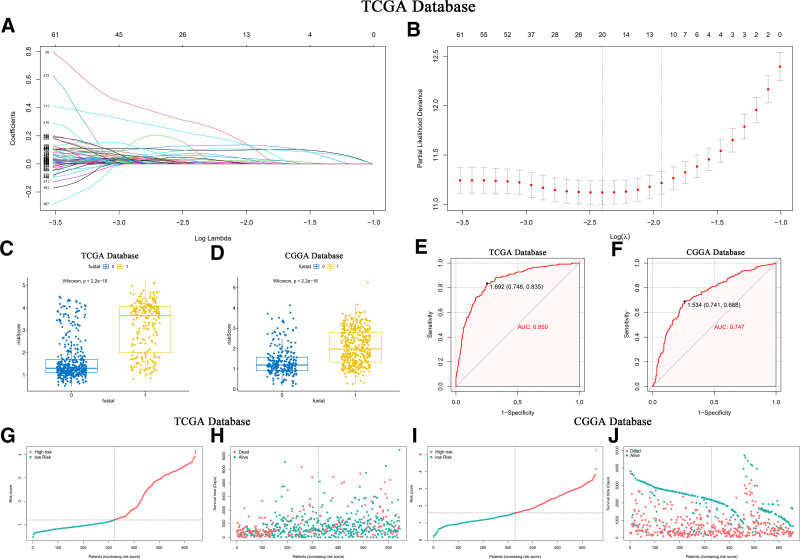
LASSO-COX analysis of glioma samples in the TCGA database with validation in the CGGA database. A and B show the LASSO-COX analysis of glioma samples from the TCGA database, where 20 candidate genes and their corresponding regression Coef were identified based on the *λ* value associated with the smallest partial likelihood deviance, facilitating the calculation of each patient’s risk score. C and D illustrate that in both the TCGA and CGGA databases, patients who survived had lower risk scores, with the significance of prognostic value assessed via the log-rank test. E and F present the ROC curves for risk scores against patient survival status, with an AUC of 0.850 for the TCGA database and 0.747 for the CGGA database. G and I depict the risk distribution plots for patients in the TCGA and CGGA databases based on risk scores, while H and J plot the risk distribution based on survival time. AUC = area under the curve, CGGA = Chinese Glioma Genome Atlas, ROC = receiver operating characteristic, TCGA = the Cancer Genome Atlas.

Division of TCGA database glioma samples by the median expression of SOCS1 into high and low groups facilitated the construction of a risk profile. This profile was developed through LASSO-COX dimensionality reduction, focusing on the differential gene responses between these SOCS1 expression groups (Fig. 12A and B). Based on the *λ* value corresponding to the smallest partial likelihood deviance, 20 candidate genes and their respective regression Coef were identified. Each patient’s risk score was calculated, with candidate genes and corresponding regression Coef for the TCGA and CGGA databases presented in Table 1. Patients were divided into high-risk and low-risk groups based on the median risk score. It was found that patients with higher risk scores had a higher mortality rate, a result that was statistically significant and validated in the CGGA database (Fig. 12C and D). The predictive capability of the risk scores was evaluated using ROC curves, revealing an AUC of 0.850 in the TCGA database (Fig. 12E) and 0.747 in the CGGA database (Fig. 12F). The correlation between patients’ risk scores, survival time, and survival status in the 2 databases was depicted, demonstrating the difference in risk scores across different survival statuses (Fig. 12G–J). These findings validate the credibility of the risk score based on differential expression of SOCS1.

**Table 1:** Gene represents candidate genes obtained from LASSO-COX dimensionality reduction analysis.

	Gene	TCGA coef	CGGA coef
1	IGFBP2	0.0951	0.0951
2	SPAG4	0.0312	0.0312
3	TNFAIP6	0.0154	0.0154
4	RAB42	0.1298	0.1298
5	EMP3	0.1040	0.1040
6	CLEC18C	0.2939	0.2939
7	POTEI	0.0940	0.0086
8	MEOX2	0.0086	0.0021
9	SAA1	0.0021	0.0126
10	ABCC3	0.0126	0.0418
11	PLAUR	0.0418	0.0185
12	FBXO39	0.0185	0.0026
13	ARL9	0.0026	0.0600
14	EN1	0.0600	0.0011
15	PRR33	0.0217	0.0776
16	RARRES1	0.0011	0.0022
17	GPR1	0.0776	0.0004
18	HOXD12	0.2105	\
19	PODNL1	0.0022	\
20	STAP1	0.0004	\

Coef represents the corresponding regression coefficients. Genes not found in the CGGA database are denoted by \. Coefficients are retained to 4 decimal places.

CGGA = Chinese Glioma Genome Atlas, Coef = coefficients.

### 3.14. Incorporating SOCS1 differential expression risk scores into the nomogram model enhances accuracy

**Figure 13.**
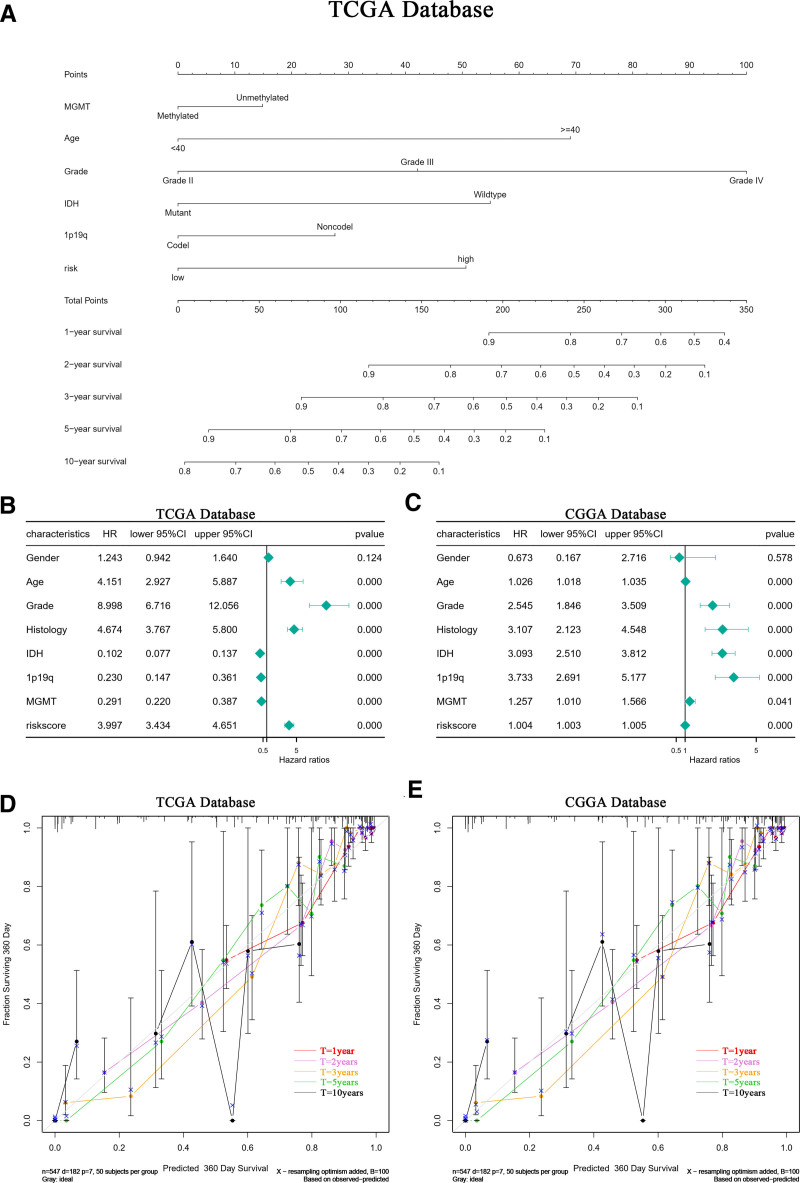
OS nomogram model for glioma patients. (A) The application of the nomogram model accurately predicts the 1, 2, 3, 5, and 10-year survival rates of glioma patients. (B and C) Multivariate analysis of prognostic factors included in the nomogram model for overall survival (OS) in the TCGA and CGGA databases. (D and E) Calibration plots illustrate the comparison between predicted OS and actual OS for 1, 2, 3, 5, and 10-year survival probabilities in the TCGA and CGGA databases. CGGA = Chinese Glioma Genome Atlas, TCGA = the Cancer Genome Atlas.

A nomogram model for predicting survival time was developed using independent prognostic factors such as risk score, MGMT methylation status, WHO grading, diagnostic age, 1p/19q co-deletion status, and IDH mutation status. This model features a scoring system at the top and a prediction system at the bottom, where individual factor scores and total scores can accurately project the survival rates for glioma patients at 1, 2, 3, 5, and 10 years (Fig. 13A). These prognostic factors were further analyzed in a multivariate Cox proportional hazards model to affirm the predictive model’s efficacy (Fig. 13B and C). Calibration curves demonstrated significant alignment between the nomogram model’s predictions and actual outcomes in both the TCGA and CGGA databases, signifying the model’s optimal predictive accuracy (Fig. 13D and E). Additionally, risk scores derived from LASSO-COX dimensionality reduction analysis of other independent prognostic factors were included in different nomogram models. The C-index values of these models were evaluated and compared to those of models constructed strictly from independent prognostic factors without LASSO-COX adjustments (see Table 2). The findings indicated that models incorporating independent prognostic factors refined through LASSO-COX analysis significantly enhance predictive accuracy. Notably, the nomogram model including SOCS1 expression difference risk score achieved a C-index of 0.862, surpassing all other predictive models.

**Table 2:** Evaluation of the predictive performance for OS using the C-index for models with and without LASSO-COX dimensionality reduction, as well as glioma clinical prognostic factors.

	PM1	PM2	Age	Grade	1p19q	IDH	MGMT	Risk
With LASSO-COX	0.862	0.75	0.667	0.779	0.615	0.795	0.663	0.762
Without LASSO-COX	0.799	0.743	0.587	0.624	0.608	0.655	\	\

PM1: prediction model (TCGA), PM2: prediction model (CGGA), with LASSO-COX: processed through LASSO-COX dimensionality reduction analysis; without LASSO-COX: not processed through LASSO-COX dimensionality reduction analysis. Clinical characteristics that did not meet the criteria for validation as independent prognostic factors in multivariate regression analysis are not included and are denoted by \.

CGGA = Chinese Glioma Genome Atlas, IDH = isocitrate dehydrogenase, OS = overall survival.

## 4. Discussion

Gliomas are malignancies originating from glial cells within the nervous system and represent common intracranial tumors, accounting for about 40% to 50% of all such tumors. Conventionally, treatments include surgery, radiotherapy, and chemotherapy, though these often yield minimal benefits.^[27]^ Tumor immunotherapy aims to elicit specific immune responses through active or passive mechanisms to suppress and eradicate tumor cells, offering benefits like specificity, efficiency, and minimal harm to the body. It shows promising clinical outcomes across most cancers. Despite suggestions to include immunotherapy in glioma treatment protocols, the efficacy of such therapies is hampered by the distinctively suppressive immune microenvironment present in higher-grade gliomas.^[28]^

In recent years, increasing evidence has suggested potential benefits of SOCS1 in cancer treatment,^[29–34]^ yet its therapeutic implications for gliomas remain unclear. This study, therefore, undertook an analysis of gene sequencing data and patient clinical information to explore and confirm the biological functions of SOCS1 in gliomas, its expression within the immune microenvironment, and its utility in prognostic predictions for patients.

Pairwise analysis of SOCS1’s expression in tumor and normal samples from the TCGA database revealed varying levels of expression across different cancers. High expression was noted in several cancers, including breast invasive carcinoma, esophageal carcinoma, and head and neck squamous cell carcinoma. Conversely, lower expression was observed in cancers such as bladder, stomach, and liver cancer. A comparison of tumor samples from the TCGA database with normal samples from the GTEx database showed that SOCS1 is more higher expression of SOCS1 in gliomas than in normal tissues. Pan-cancer analysis conducted with the TCGA database demonstrated a positive correlation between SOCS1 expression and TMB and MSI in certain cancers, and a significant positive correlation with immune scores in most cancers, including gliomas. Further investigation into SOCS1’s expression in gliomas, using single-cell sequencing data from the CGGA and GEO databases, indicated that SOCS1 predominantly accumulates in tumor cells and T cells, exhibiting a similar expression pattern to immune markers such as CD8A and IL7R.

To explore the biological functions of SOCS1 in glioma, differential expression genes of SOCS1 in the TCGA database were first subjected to GO and KEGG pathway enrichment. Then, through the Pearson algorithm, the top 203 genes most closely associated with SOCS1 from the differential expression gene sets in the CGGA and TCGA databases were selected for GO, KEGG, and GSEA enrichment analyses, indicating that SOCS1 may be involved in tumor immunity, inflammatory responses, as well as in the proliferation, differentiation, and apoptosis of T cells. To validate this association, the correlation between SOCS1 and 8 immune checkpoints, as well as 9 clusters of genes related to the immune system or inflammatory responses in the CGGA and TCGA databases, was examined through the Pearson algorithm, showing that SOCS1 shares similar expression patterns and roles in inflammatory responses with most immune checkpoints and exhibits significant positive correlation with most metagene clusters. Pearson correlation analysis between SOCS1 expression and enrichment scores of 13 immune or inflammatory functions obtained through GSVA analysis in the CGGA and TCGA databases revealed that SOCS1 expression is linked to the proliferation and apoptosis of T cells and CD8 in glioma.

To explore the expression of SOCS1 within the glioma immune microenvironment, we investigated its correlation with glioma immune scores. Our analysis revealed that elevated SOCS1 expression is associated with increased immune scores and reduced tumor purity, similar to most immune checkpoints. The degree of immune cell infiltration and enrichment scores in high and low SOCS1 expression groups were assessed using ssGSEA and Xcell, revealing higher immune cell infiltration and enrichment scores in the high SOCS1 expression group. Immune cell abundance and the proportion of 22 types of immune cells in glioma samples from the CGGA and TCGA databases were estimated using CIBERSORT, with the highest ratio scores found in macrophages M2, followed by T cells CD8. Additionally, SOCS1 exhibited a positive correlation with most T cells, NK cells, and macrophages. Comparing the correlation between 9 immune checkpoints and 22 types of immune cells, CTLA4 showed the most similar results to SOCS1, which also shared similarities with other immune checkpoints. Analysis of glioma mutation data from the TCGA database indicated that the high SOCS1 expression group had a higher tumor mutation burden to some extent. Testing for immune checkpoint blockade responses with 3 immune checkpoints, PDCD1, CD274, and PDCD1LG2, revealed a higher blockade response in the high SOCS1 expression group. Drug sensitivity analysis of 2 classical antitumor drugs showed lower IC50 values for the high SOCS1 expression group, suggesting potentially better drug sensitivity.

Through the analysis of clinical features of 1343 glioma patients from the TCGA and CGGA databases, SOCS1 emerged as a promising marker for the prognosis and prediction of high-grade gliomas. Evaluations using ROC, time ROC, and Kaplan–Meier curves assessed SOCS1’s effectiveness in discerning and forecasting glioma patients’ pathological characteristics and survival outcomes. A univariate Cox regression analysis that included SOCS1 alongside 9 immune checkpoints revealed SOCS1’s hazard ratio to be higher than most of the checkpoints, marking significant distinctions. Further analysis, incorporating SOCS1 and clinical features of glioma patients into both univariate and multivariate Cox regression models, established SOCS1 as a positive, independent prognostic factor.

Risk scores, derived from differential genes associated with SOCS1 expression levels, were calculated using LASSO-COX dimensionality reduction analysis. The prognostic prediction capability of these risk scores for glioma patients was assessed through comparative analyses and ROC curves, depicting the patients’ risk distribution based on their scores and survival times. In the TCGA database, a nomogram model that integrated these risk scores and independent prognostic factors for glioma patients was validated through multivariate Cox regression analysis. The model’s prediction accuracy was evaluated using calibration curves and a C-index value, revealing an excellent alignment with calibration curves and achieving a C-index of 0.862, surpassing other predictive models. This finding was corroborated in the CGGA database.

In summary, the biological function of SOCS1 in glioma, its expression within the immune microenvironment, and its correlation with immune cells are similar to most immune checkpoints. This suggests that SOCS1 could serve as a potential target for immunotherapy in glioma patients. Our validation of its potential therapeutic effects revealed that the high SOCS1 expression group, because of higher blockade responses and better drug sensitivity, could respond more favorably to immunotherapy. Furthermore, risk scores using a differential expression of SOCS1 as a positive regulatory risk feature demonstrate high predictive accuracy and validity, significantly enhancing the precision of diagnostic and prognostic models for glioma patients.

This study also suggests that immunotherapy, particularly inhibitors targeting the CTLA4 immune checkpoint, has significantly improved treatment outcomes for patients with advanced malignant tumors.^[35,36]^ The relationship between SOCS1 and immune cells in glioma, which closely mirrors CTLA4 and the higher tumor mutation burden indicated by high SOCS1 expression, suggests a substantial therapeutic potential of SOCS1 in glioma.

Nonetheless, there are limitations to this study. The scarcity of public single-cell sequencing data from glioma patients makes it challenging to provide multi-sample single-cell analysis validation. Furthermore, the limited number of normal samples paired with glioma patient tumor samples does not allow for further elucidation of SOCS1 expression differences. Lastly, the absence of follow-up experiments to validate the conclusions. Therefore, the next steps of this research will involve designing basic experiments, establishing an independent single-cell sequencing database for glioma patients, and extracting more paired normal samples for improvement.

## Acknowledgments

We thank Home for Researchers editorial team (www.home-for-researchers.com) for language editing service.

## Author contributions

**Conceptualization:** Chuanshen Gu, Xinyi Chen.

**Data curation:** Jiayan Wu, Yiwen Zhang.

**Formal analysis:** Linyu Zhong, Han Luo.

**Funding acquisition:** Wenshu Luo.

**Software:** Chuanshen Gu, Xinyi Chen.

**Validation:** Jiayan Wu.

**Writing – original draft:** Yiwen Zhang, Linyu Zhong, Han Luo.

**Writing – review & editing:** Wenshu Luo, Fuxia Yang.
